# Investigation of Effect of Preliminary Annealing on Superplasticity of Ultrafine-Grained Conductor Aluminum Alloys Al-0.5%Mg-Sc

**DOI:** 10.3390/ma15010176

**Published:** 2021-12-27

**Authors:** Mikhail Gryaznov, Sergey Shotin, Aleksey Nokhrin, Vladimir Chuvil’deev, Constantine Likhnitskii, Vladimir Kopylov, Mikhail Chegurov, Nataliya Tabachkova, Iana Shadrina, Elena Smirnova, Olga Pirozhnikova

**Affiliations:** 1 Materials Science Department, Physical-Technical Research Institute, Lobachevsky State University of Nizhniy Novgorod, 603022 Nizhny Novgorod, Russia; gryaznov@nifti.unn.ru (M.G.); shotin@nifti.unn.ru (S.S.); chuvildeev@nifti.unn.ru (V.C.); constantine.lkv@yandex.ru (C.L.); kopylov@nifti.unn.ru (V.K.); mkchegurov@nifti.unn.ru (M.C.); yashadrina@nifti.unn.ru (I.S.); smirnova@nifti.unn.ru (E.S.); opiro@mail.ru (O.P.); 2 Laboratory of Vacuum Plasma Coating, Physical-Technical Institute, National Academy of Sciences of Belarus, 220141 Minsk, Belarus; 3 Center Collective Use “Materials Science and Metallurgy”, National University of Science and Technology “MISIS”, 119991 Moscow, Russia; ntabachkova@misis.ru; 4 Laboratory “FIANIT”, Laser Materials and Technology Research Center, A.M. Prokhorov General Physics Institute, Russian Academy of Sciences, 119991 Moscow, Russia

**Keywords:** aluminum alloys, scandium, ultrafine-grained structure, superplasticity, dynamic grain growth, cavitation

## Abstract

Effect of preliminary precipitation of Al_3_Sc particles on the characteristics of superplastic conductor Al-0.5%Mg-X%Sc (X = 0.2, 0.3, 0.4, 0.5 wt.%) alloys with ultrafine-grained (UFG) microstructure has been studied. The precipitation of the Al_3_Sc particles took place during long-time annealing of the alloys at 300 °C. The preliminary annealing was shown to affect the superplasticity characteristics of the UFG Al-0.5%Mg-X%Sc alloys (the elongation to failure, yield stress, dynamic grain growth rate) weakly but to promote more intensive pore formation and to reduce the volume fraction of the recrystallized microstructure in the deformed and non-deformed parts of the aluminum alloy specimens. The dynamic grain growth was shown to go in the deformed specimen material nonuniformly–the maximum volume fraction of the recrystallized microstructure was observed in the regions of the localization of plastic deformation.

## 1. Introduction

At present, microdoped high-strength Al alloys are considered to be promising materials for electrical engineering, in particular, for the replacement of copper alloys in small-sized avionics wiring by the Al alloys [1,2,3]. It will allow reducing the weight of the on-board wiring of modern aircraft and increasing the load capacity, energy efficiency, etc., of these ones in the future. The conductor Al alloys should have high strength and thermal stability [1,2,3] as well as good plasticity at room and elevated temperatures to ensure the possibility of making small-sized bimetallic wires of 0.2–0.5 mm in diameter by drawing or rolling from workpieces.

The Al alloys microalloyed with additives of rare earth elements (REEs) and of transition metals (TMs)–Sc, Zr, Hf, etc., are promising conductor materials [4,5,6,7,8,9,10,11,12]. Nucleation of Al_3_(REE,TM) nanoparticles with Ll_2_ structure allows providing a high level of the thermal stability of the ultrafine-grained (UFG) microstructure in the severely deformed Al alloys [13,14,15,16,17,18,19,20,21,22,23] and, as a consequence, high superplastic characteristics of these ones [24,25,26,27,28,29,30]. The studies on the superplasticity characteristics of the UFG conductor aluminum alloys with ultra-low Mg content are almost absent. There are few papers on the superplasticity of UFG conductor 6061 aluminum alloys [31,32,33] and UFG Al-1%Zr alloys [34]. Numerous papers demonstrated the reduction of the grain sizes *d* down to nano- and submicron scale to result in higher strength, hardness, and fatigue resistance of the Al-Mg-Sc alloys [5,12,15,16,18,19,35,36,37].

It was shown in [30] that intensive pore formation in the Al_3_Sc incoherent particles may be one of the factors limiting the ultimate superplastic characteristics of the UFG Al-0.5%Mg-Sc alloys. It should be stressed here that the formation of the Al_3_Sc particles in the investigated UFG alloys at reduced temperatures goes via a discontinuous precipitation mechanism (see [4,30,38]) similar to the low-temperature discontinuous solid solution decomposition in Al-Zr alloys [39,40,41,42,43,44,45,46,47,48,49].

The micropore formation in large Al_3_Sc particles may be related to the damping of cutting of such incoherent particles by lattice dislocations and disclination-type defects will form in the course of superplastic deformation [44,45]. Such a defect forming at an incoherent particle with the radius R located inside the grain boundary during the deformation in the first approximation can be described as a disclination loop with the radius R and power *ω*(*τ*), the magnitude of which grows proportionally to the number of defects occurring at the grain boundary [44,45]. As it has been shown in [44,45], at certain critical power ω*, the excess energy of the disclination loop becomes so high that it becomes energetically favorable for the grain boundary to “release” from the source of this latent energy. At the elevated test temperatures, such a “relaxation” of the accumulated energy may take place through the micropore formation at the interphase boundaries “Al_3_Sc particle–Al” [30]. In the course of the superplastic deformation, such micropores can become the origins of cavitation fractures in the UFG Al-0.5%Mg-Sc alloys [30].

The present papers were aimed at an additional verification of the hypothesis proposed in [30] on the effect of the Al_3_Sc particles on the superplastic characteristics of the UFG Al-0.5%Mg-Sc alloys. Note that the intermittent decomposition of solid solution leads to the formation of large dashed linewise Al_3_(REE,TM) incoherent particles at the grain boundaries in the Al alloys (see [30,39,40]). It may lead to an increased number of failures of the small-sized wires of 0.2–0.3 mm in diameter in the course of fabrication by drawing, rolling, or extraction. In this connection, the issue considered is of high practical importance for solving the problem of choice of the optimal thermal treatment regimens of the UFG Al-0.5%Mg-Sc alloys prior to fabricating the small-sized wires.

## 2. Materials and Methods

The Al-0.5 wt.%Mg alloys with different Sc contents (0.2, 0.3, 0.4, and 0.5 wt.%Sc) were the objects of investigation. The alloy specimens 22 × 22 × 150 mm in sizes were obtained by induction casting in INDUTHERM^®^ VTC-200 casting machine (Indutherm GmbH, Walzbachtal, Germany) according to the procedure described in [30,38]. After casting, the alloys were not subjected to homogenization. The UFG structure in the workpieces was formed by Equal Channel Angular Pressing (ECAP) using Ficep^®^ HF 400 L hydraulic press (Ficep^®^ S.P.A., Varese, Italy) by the modes: temperature T_ECAP_ = 225 °C, strain rate 0.4 mm/c, number of cycles–N = 4, ECAP regime–B_c_. The warm-up time of the workpiece prior to ECAP was 10 min, the holding time of the UFG workpiece in the instrumentation after ECAP did not exceed 5 min.

The mechanical tension testing of the flat double-blade shaped specimens with working parts 3 mm long and 2 × 2 mm in cross-sections was carried out using Tinius Olsen H25K-S tension machine (Tinius Olsen Ltd., Surrey, UK). Testing was performed in the temperature range from 300 to 500 °C; the tension rate varied from 10^−3^ to 3.3 × 10^−1^ s^−1^. The holding time of the specimen in the furnace prior to the superplasticity experiments was 5 min. The uncertainty of the temperature maintenance during the superplasticity tests was ±5 °C. The temperature was measured by a thermocouple placed as close as possible to the specimen clamping area. During the experiment, the “stress (*σ*)–strain (*ε*)” curves were acquired, which the values of the relative elongation to failure (*δ*) and of the yield stress (*σ_b_*) were determined from.

**Figure 1:**
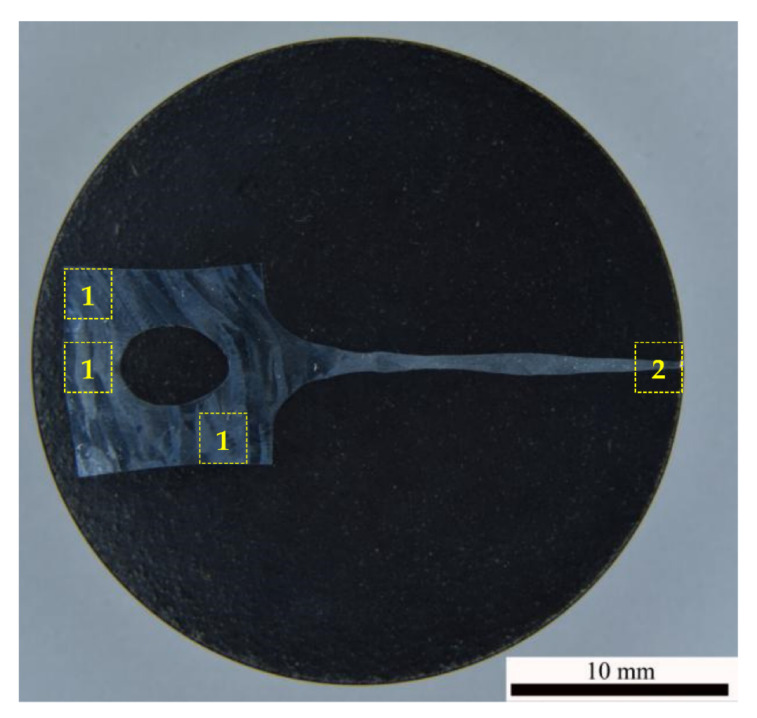
General view of a specimen of UFG alloy Al-0.5%Mg-0.5%Sc after the superplasticity testing (500 °C, 10^−2^ s^−1^). The areas of investigation of the grain microstructure and measuring the microhardness in the non-deformed and deformed parts are marked as (1) and (2), respectively. In the non-deformed part, the dendrite boundaries are visible, inside which a uniform UFG microstructure was formed.

Chemical analysis was performed using iCAP^®^ 6300-ICP-OES Radial View™ spectrometer with induction-coupled plasma (Thermo Scientific, Waltham, MA, USA). To study the macro- and microstructure of the alloys, a Leica^®^ IM DRM metallographic optical microscope (Leica Microsystems GmbH, Wetzlar, Germany), a Jeol^®^ JSM-6490 Scanning Electron Microscope (SEM), and a Jeol^®^ JEM-2100 Transmission (TEM) were used (Jeol Ltd., Tokyo, Japan). To study the macro- and microstructure, the specimen surfaces were subjected to mechanical grinding with diamond pastes to the roughness <1 μm followed by polishing in 8%HClO_4_ + 9%H_2_O + 10%C_6_H_14_O_2_ + 73%C_2_H_5_OH solution. The microstructure was revealed by etching in a glycerin-based solution (1%HF + 1.5%HCl + 2.5%HNO_3_ + 95% glycerine); the macrostructure-by etching in 40%HNO_3_ + 40%HCl + 20% HF solution. The mean grain sizes (*d*) and the volume fraction of the recrystallized microstructure (*f_R_*) were determined using GoodGrains software (UNN, Nizhny Novgorod, Russia). The mean sizes of the Al grains and Al_3_Sc particles were determined by the chord method. The microhardness (*H_v_*) measurements were performed using an HVS1000 hardness tester (INNOVATEST Europe BV, Maastricht, The Netherlands). The areas of the microstructure and microhardness areas are marked by yellow dashed lines in Figure 1.

The fractographic analysis of the fractures was carried out using Jeol^®^ JSM-6490 SEM. The analysis of the specimen fractures was carried out according to the classification described in [43].

The microstructure and microhardness measurements on the specimens after the superplasticity testing were performed in two areas–in the non-deformed area of the specimen (Zone I) and in the deformed one, the closest to the destruction center (Zone II). For the investigations, the specimens were pressed into a WEM REM mixture (Cloeren Technology GmbH, Berlin, Germany) and subjected to mechanical grinding, electrochemical polishing, and wet chemical etching according to the procedure described above. In the case when the destruction area was not in the center of the tested specimen, the part of the specimen, which has been subjected to the highest tensile strain in the course of testing was selected for the microstructure and fractographic analysis.

The annealing of the specimens was performed in an EKPS-10 air furnace (Smolensk SKTB SPU JSC, Smolensk, Russia). The uncertainty of the temperature maintenance in the furnace was ±10 °C. After annealing, the specimens were cooled down in the air.

## 3. Results

### 3.1. Microstructure Investigation

**Figure 2:**
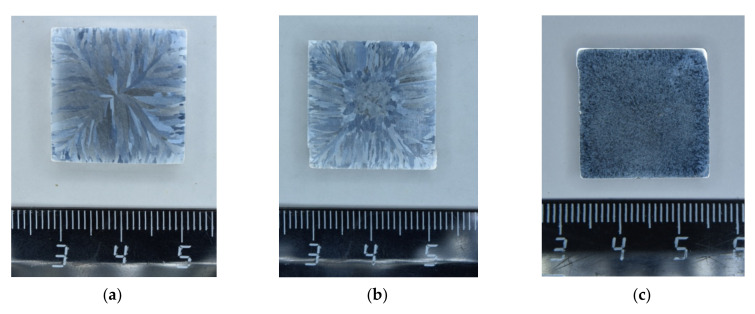

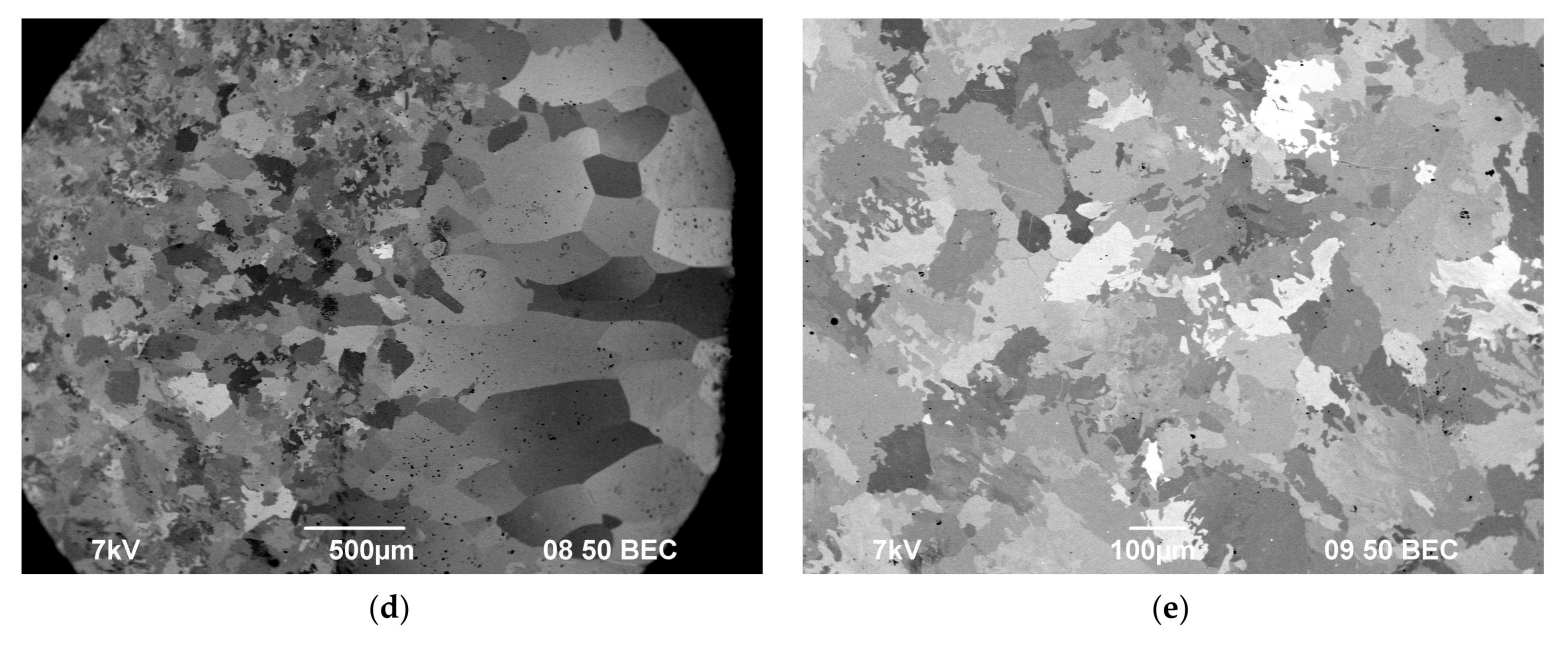
Macrostructure of the cast Al-0.5%Mg-Sc alloy with different Sc contents: (**a**) 0.2%Sc; (**b**) 0.3%Sc; (**c**) 0.5%Sc [38]. (**d**)—the edge, (**e**)—the center of the Al-0.5%Mg-0.5%Sc alloy bulk. The cross-section of the bulk sample.

The Al-0.5%Mg-Sc cast alloys had a dendrite-wise coarse-grained macrostructure: columnar crystals in the rapid cool-down zone at the specimen edges and equiaxial grains in the central parts of the bulks (Figure 2). The polished area occupied by the equiaxial grains increased with increasing Sc content. In the alloy with 0.5%Sc, a uniform macrostructure was formed (Figure 2c) consisting of the grains with nearly equiaxial shapes almost completely. At the sides of the Al-0.5%Mg-0.5%Sc bulk, there were large equiaxial grains of ~0.5 mm in size. The residual dendrite macrostructure in the alloy with 0.5%Sc was observed in the upper part of the bulk only. The average grain sizes in the central parts of the bulks decreased from 1.0–1.2 mm (Figure 2a) down to 30–100 μm with increasing Sc content from 0.2 up to 0.5% (Figure 2e). The porosity of the central parts of the bulks was absent.

**Figure 3:**
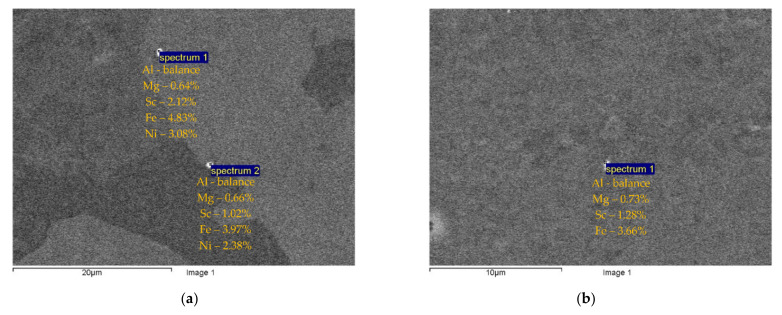
Al_3_Sc primary particles in the cast (**a**) and UFG alloy (**b**) Al-0.5%Mg-0.5%Sc. SEM.

The microstructure investigations have shown large light-colored micron-sized particles consisting of Sc and Al only in the cast alloys with 0.4 and 0.5%Sc (Figure 3). According to [4,13,14,15,16,17], these are probably Al_3_Sc particles. Primary Al_3_Sc particles contain Fe and Ni in their composition (Figure 3). The particles are distributed uniformly enough inside the bulk; an insufficient increase of the volume fraction of the primary particles in the central parts of the bulks of the alloys with 0.4% and 0.5%Sc was observed.

**Figure 4:**
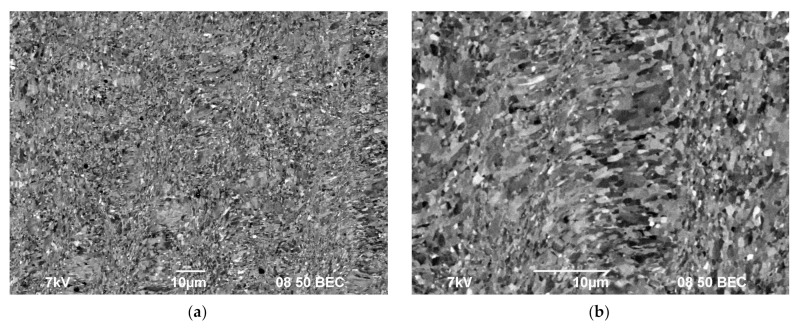
Macrostructure (**a**) and microstructure (**b**) of the UFG Al-0.5%Mg-0.5%Sc alloy after ECAP. SEM.

In the metallographic investigations, the macrostructure of the UFG alloys after four cycles of ECAP (T_ECAP_ = 225 °C) comprises the crossing microbands of localized plastic deformation (Figure 4a). The presence of the localized deformation strips affects the grain morphology in the areas of the bands crossing (Figure 4b).

**Figure 5:**
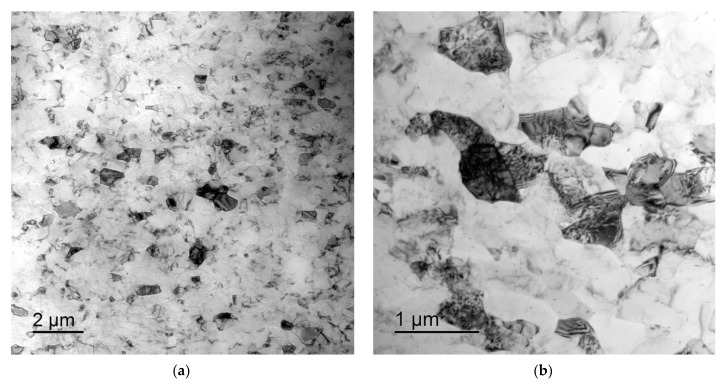

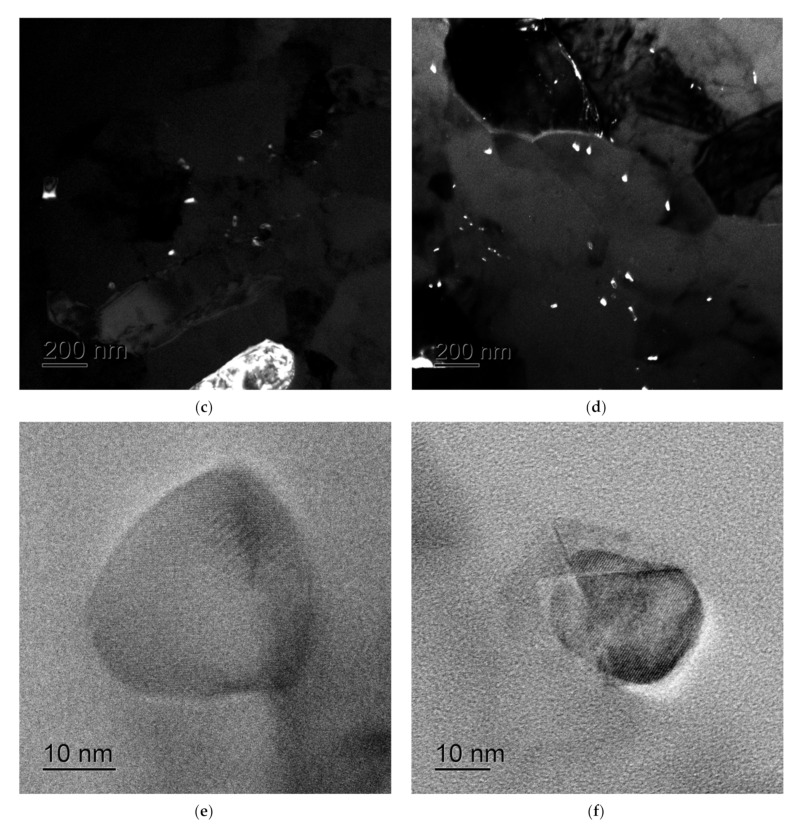
Microstructure of the UFG Al-0.5%Mg-0.2%Sc (**a**,**c**,**e**) and Al-0.5%Mg-0.5%Sc alloys (**b**,**d**,**f**) after ECAP: (**a**,**b**) bright-field images of the microstructure of UFG alloys; (**c**,**d**) dark field images of the Al_3_Sc particles c; (**d**,**f**) bright field images of the Al_3_Sc nanoparticles. TEM.

After ECAP, a uniform UFG structure formed, the average grain sizes were 0.4–0.6 μm and almost did not depend on the Sc concentration (see Figure 5a,b). There were no abnormally large grains in the UFG alloys after ECAP (Figure 4b and Figure 5a,b). In the UFG alloys, there were few submicron Al_3_Sc particles (Figure 5c,d). An insufficient increase of the volume fraction of the primary Al_3_Sc particles with increasing Sc content was observed (Figure 5c,d); the particle sizes almost did not change. The parameters (the sizes, the quantity, the positions inside the workpiece) and the composition of the particles in the cast and UFG alloys were close to each other (Figure 3). It allows suggesting that the submicron Al_3_Sc particles observed in the UFG alloys form during the bulk crystallization. The Al_3_Sc particles were coherent to the Al crystal lattice: the elastic strain fields were observed near the particles while the interphase boundaries between the Al_3_Sc particles and the Al crystal lattice were diffused (Figure 5e,f, see also [38]). No large elongated Al_3_Sc particles, the presence of which evidences the intermittent mechanism of the particle nucleation (see [39,40,41,42,43,44,45,46,47,48,49]) were found.

According to [30,38], the temperature of the recrystallization during 30-min annealing of the UFG Al-0.5%Mg-Sc alloys is ~350–375 °C. The high thermal stability of the UFG structure of the alloys originates from the early nucleation of the Al_3_Sc particles. In [38], the dependence of the specific electrical resistivity (SER) on the annealing temperature has been investigated. From the analysis of the results, the solid solution decomposition in the UFG Al-0.5%Mg-Sc alloy was shown to begin at 200–225 °C and to almost complete after 30-min annealing at 325–375 °C. Heating up to temperatures over 425 °C results in an increase of the SER, obviously, due to the dissolving of the Al_3_Sc particles nucleated earlier.

**Figure 6:**
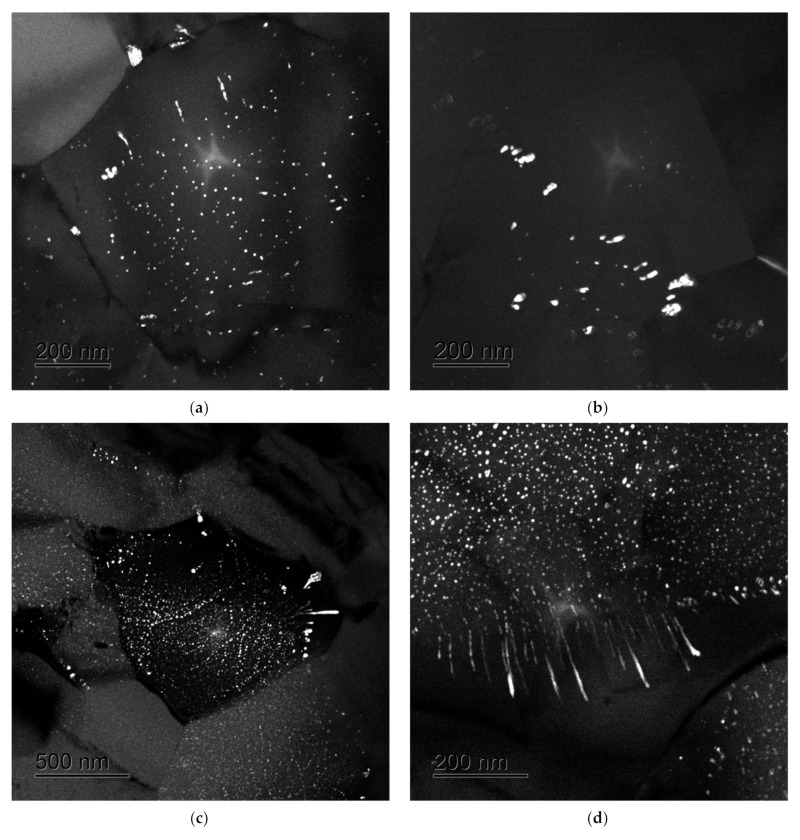
Nucleation of the Al_3_Sc particles in the UFG Al-0.5%Mg-Sc alloys after long-time annealing at 300 °C: (**a**,**b**) Al-0.5%Mg-0.2%Sc; (**c**) Al-0.5%Mg-0.3%Sc; (**d**) Al-0.5%Mg-0.4%Sc.

**Figure 7:**
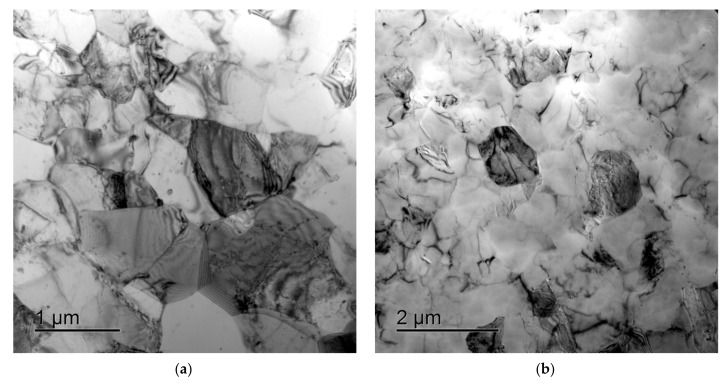
Microstructure of the UFG Al-0.5%Mg-Sc alloys after long-time annealing at 300 °C: (**a**) Al-0.5%Mg-0.3%Sc; (**b**) Al-0.5%Mg-0.4%Sc.

According to the TEM data, several types of Al_3_Sc particles are formed in the UFG Al-0.5%Mg-Sc alloys in the course of long-time annealing (up to 300 h) at 275–300 °C. In the annealed UFG alloy, coherent nanoparticles of 10–30 nm in sizes nucleated inside the grains and at the grain boundaries were observed, as well as large dashed-linewise Al_3_Sc particles of 50–200 nm in sizes nucleated near the grain boundaries via the discontinuous precipitation mechanism (Figure 6). It should be stressed here that the mean grain sizes almost did not change during annealing, and the annealed alloys preserved the UFG microstructure (Figure 7). The results of investigations by electron microscopy presented in Figure 6 supported the conclusion made in [38] on the two-stage character of the Al_3_Sc particle nucleation during annealing the UFG Al-0.5%Mg-Sc alloys. In [38], on the basis of analysis of the results of investigations of the dependence of the specific electrical resistivity on the annealing time, the two-stage character of the Al_3_Sc particle nucleation was shown to originate from the competition of the grain boundary diffusion (small holding times) and diffusion along the lattice dislocation cores (large isothermic holding times and/or elevated annealing temperatures).

**Figure 8:**
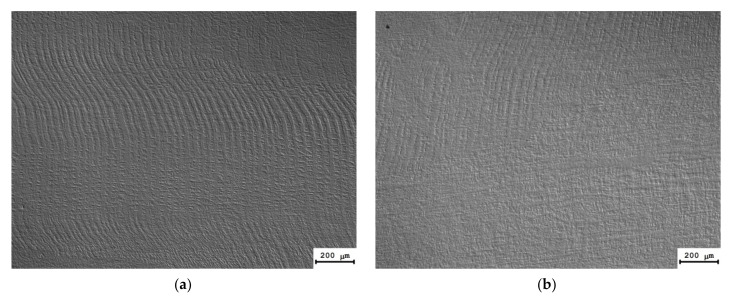
Macrostructure of the UFG Al-0.5%Mg-Sc alloys after long-time annealing at 300 °C: (**a**) Al-0.5%Mg-0.2%Sc; (**b**) Al-0.5%Mg-0.3%Sc.

As has been noted above, the microstructure of the UFG alloys comprises a site of crossing localized strain bands (Figure 4a). The metallographic investigations have shown the deformation “banding” formed during ECAP to reproduce well in the microstructure of the UFG alloys after long-time holding at 300 °C. As one can see in Figure 8, the localized strain bands were seen clearly after preliminary low-temperature annealing and electrochemical polishing. It is interesting to note that the low-temperature pre-recrystallization annealing did not affect the mean spacing between the bands, which was ~20–30 μm but allowed revealing these ones more clearly as compared to the UFG state after ECAP.

As has been mentioned above, the recrystallization in UFG Al-0.5%Mg-Sc alloys begins after heating up to ~350 °C (30 min). No deformation bands were found in the completely recrystallized alloys after annealing at 450–500 °C (see [38]); a uniform fine-grained structure was formed in the alloys. The mean sizes of the recrystallized grains (*d_R_*) and the volume fraction of the recrystallized material (*f_R_*) decreased with increasing Sc content. After annealing at 500 °C (30 min), the increasing of Sc content from 0.2% up to 0.5% resulted in a decreasing *f_R_* from ~100% down to ~60–70% and to a decreasing of *d_R_* from ~250 μm down to ~5 μm (see [38]). No abnormally large, recrystallized grains were observed in the UFG alloys after long-time annealing at 300 °C (Figure 8).

For the superplasticity tests, the specimens of the cast and UFG Al-0.5%Mg-Sc alloys were annealed at 300 °C for various times. The annealing was aimed at the forming of a uniform fine-grained structure with the maximum volume fraction of nucleated Al_3_Sc particles in the UFG alloys. The annealing time for each alloy was selected on the basis of the results of investigations of the solid solution decomposition presented in [38]. The annealed cast specimens of the cast and UFG Al-0.5%Mg-Sc alloys were tested for superplasticity. The results of the testing were compared to the data for the non-annealed alloys presented in [30].

### 3.2. Superplasticity Testing

#### 3.2.1. Cast Alloys

**Figure 9:**
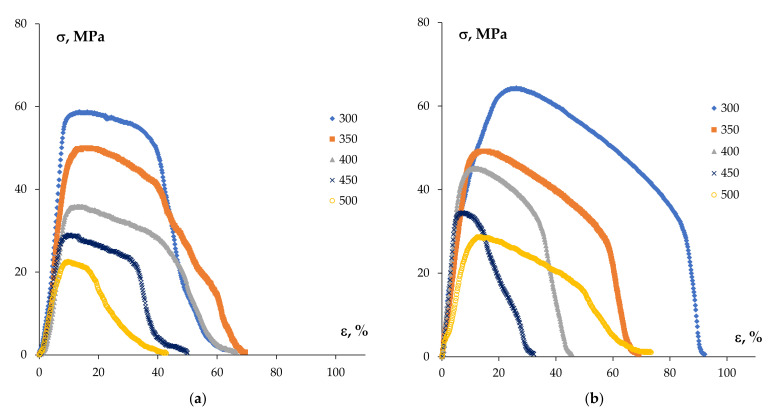
Tension curves *σ*(*ε*) for the cast Al-0.5%Mg-0.5%Sc alloys: (**a**) alloys in the initial stage; (**b**) alloys after long-time annealing at 300 °C (300 h).

As an example, the tension curves *σ*(*ε*) for the specimens of some cast alloys in the initial state and of the ones subjected to preliminary at 300 °C are presented in Figure 9. As one can see in Figure 9a, the tension curves *σ*(*ε*) of the coarse-grained Al-0.5%Mg-Sc alloys had classical three-stage character typical enough for the tension of highly plastic alloys: a short stage of the strain hardening transforming into a long stage of stable plastic flow, and, finally, the stage of localized plastic deformation finishing by the destruction of the specimen. The values of the yield stress *(σ_b_*) and of the relative elongation to failure (*δ*) are presented in Table 1. As one can see in Figure 9a and from Table 1, the temperature of testing does not affect the shapes of the *σ*(*ε*) curves considerably. The yield stress and elongation to failure decrease with increasing test temperature from 300 up to 500 °C: the values of *σ_b_* decreased from 70 MPa down to 29–30 MPa and *δ* decreased from ~62–64% down to ~43% (Table 1).

In our opinion, the reduction of *δ* in the cast alloys is related to the blocking of the lattice dislocation motion by the Al_3_Sc particles. At the same time, the Al_3_Sc particles nucleated earlier, dissolving partly with an increasing temperature that leads to an increase of the specific electrical resistance of the alloys (see [38]). The decreasing of the volume fraction and the coalescence of the Al_3_Sc particles may reduce the effect of these ones on the tensile behavior of the cast Al-0.5%Mg-Sc alloys at elevated temperatures. This leads to an insufficient increase of the elongation of the cast Al-0.5%Mg-Sc alloys at 500 °C (as compared to the one at 450 °C) again.

Long-time annealing of the cast Al-0.5%Mg-Sc alloys at 300 °C resulted in changes in the shapes of the *σ*(*ε*) curves. As one can see in Figure 9b, the stable plastic flow stages in the *σ*(*ε*) curves were absent, the stages of strain hardening transformed into one of the plastic strain localization directly.

**Figure 10:**
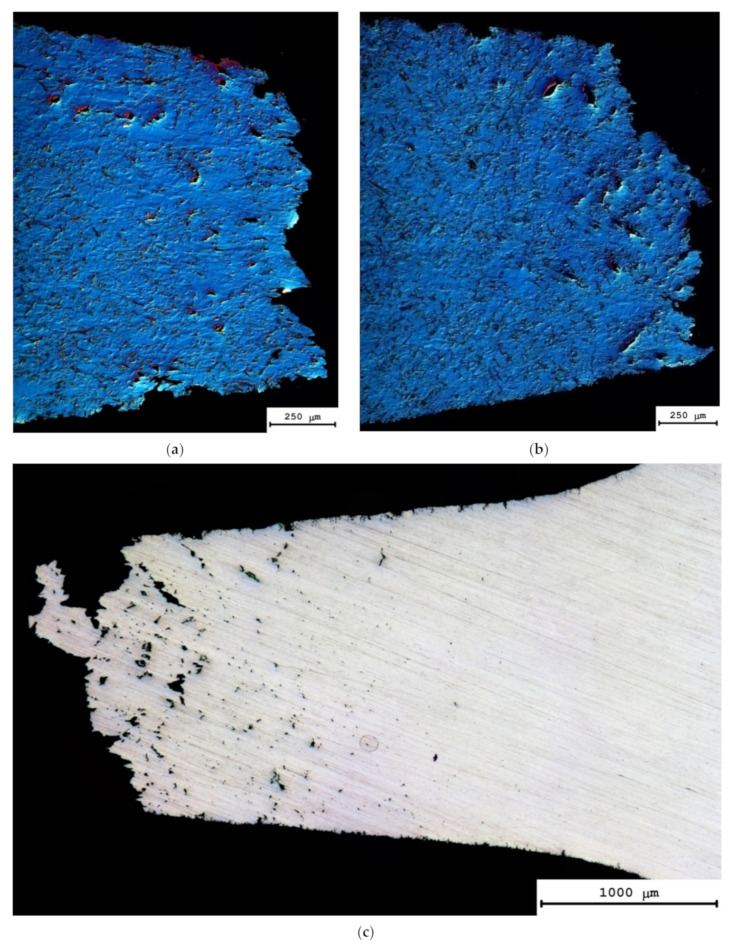
General view of the surfaces of the cast Al-0.5%Mg-0.5%Sc alloy specimens after the tension tests at 300 °C (**a**), 350 °C (**b**) and 500 °C (**c**). Metallographic optical microscopy.

The results of the metallographic investigations evidenced the formation of large pores of several tens of microns in size in the destruction region (Figure 10a). The large pores were located along the dendrite boundaries whereas the small ones–both along the grain boundaries and inside the grains. The sizes and the volume fraction of the pores decreased with increasing distance from the place of destruction. At the testing temperature of 500 °C, the zone of intensive pore formation was ~1.5–2 mm from the destruction region (Figure 10b). No pore formation was observed in the non-deformed region.

**Figure 11:**
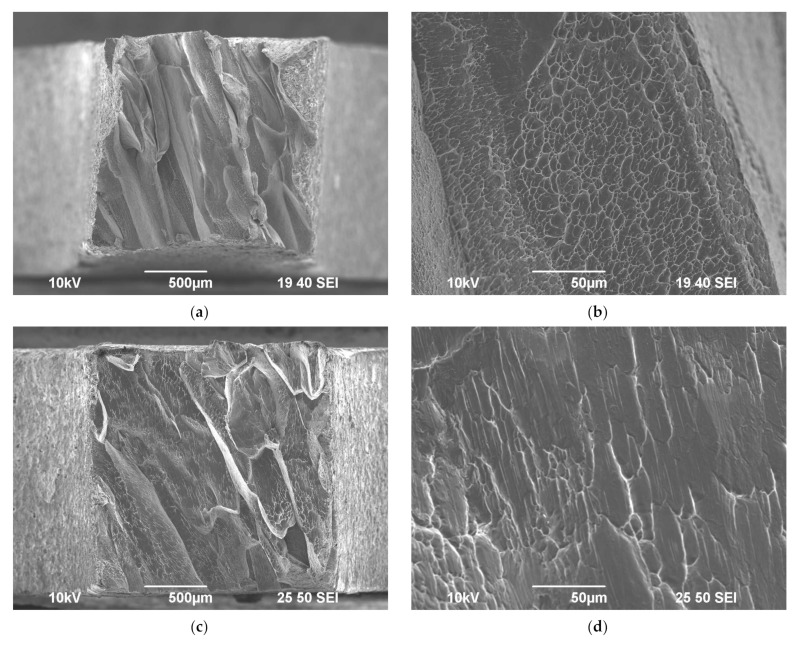

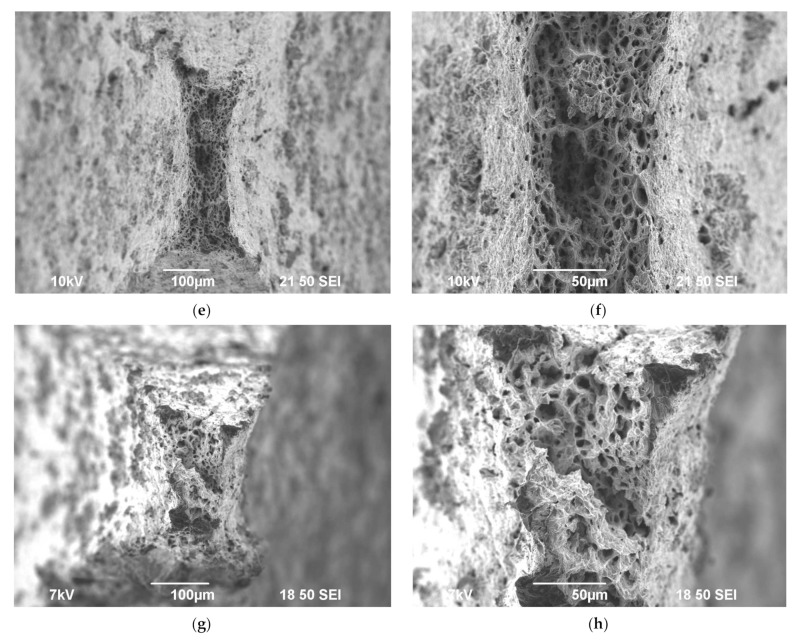
Fractographic analysis of the fractures of the specimens of the cast (**a**–**d**) and UFG (**e**–**h**) Al-0.5%Mg-0.5%Sc alloys after the tension tests at 300 °C (**a**,**b**,**e**,**f**) and 500 °C (**c**,**d**,**g**,**h**) SEM.

The results of the fractographic analysis of the fractures of the cast Al-0.5%Mg-Sc alloy specimens after the tension testing at elevated temperatures are presented in Figure 11a,c. At the macroscopic level, the specimen fractures were of the same type and comprised large shear elements, the directions of which coincided with one of the dendrite grains (see Figure 2 in [38]). At the vertices of the shear elements, the pits of various geometries were observed evidencing a viscous nature of the destruction of cast alloys (Figure 11). The variations of the deformation temperature and rate did not affect the general fracture pattern of the cast Al-0.5%Mg-Sc alloy specimens (Figure 11a,c). From the comparison of Figure 11b,d, one can see the increase of the deformation temperature to result in an increase of the pit sizes in the destruction zone of the cast alloy specimen; after testing at elevated temperatures, the pits had strongly elongated shapes (Figure 11d).

#### 3.2.2. UFG Alloys

**Figure 12:**
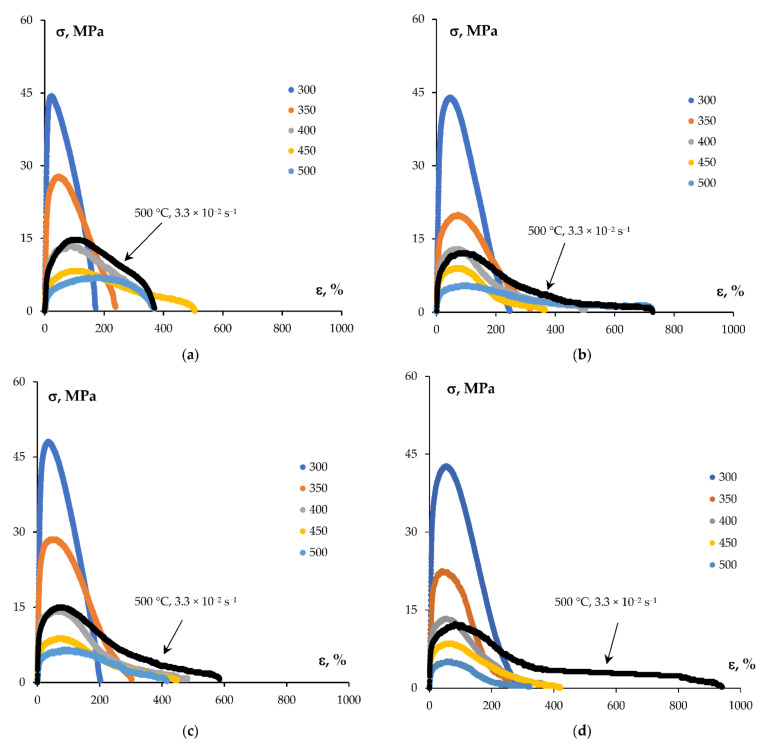
Tension curves *σ*(*ε*) for the UFG Al-0.5%Mg-Sc alloys after long-time annealing at 300 °C: (**a**) Al-0.5%Mg-0.2%Sc; (**b**) Al-0.5%Mg-0.3%Sc; (**c**) Al-0.5%Mg-0.4%Sc; (**d**) Al-0.5%Mg-0.5%Sc. Strain rate 10^−2^ s^−1^. Dark line—500 °C, 3.3 × 10^−2^ s^−1^.

Figure 12 presents the *σ*(*ε*) curves for the specimens of UFG alloys with different Sc contents. Table 2 presents the values of *σ_b_* and *δ* for the alloys investigated at various temperatures and strain rates. The *σ*(*ε*) curves acquired at 300 and 350 °C were typical enough for severely deformed metals–short stages of intensive strain hardening followed by the rapid softening were observed in the *σ*(*ε*) curves. At higher test temperatures (400–500 °C), an increase in the duration of the uniform elongation stage was observed, which reached ~200% for the UFG Al-0.5%Mg-0.2%Sc alloy at 500 °C. The degree of uniform strain decreased slightly with increasing Sc content and did not exceed 80% for the UFG Al-0.5%Mg-0.5%Sc alloy. However, a considerable general increase in the elongation to failure was observed (see Table 1). As in the case of the cast alloys, the increase of the test temperature resulted in a decrease in the yield stress but the plasticity of the UFG alloys increased essentially (Figure 12, Table 2). The dependencies *δ*(*T*) and *δ*($\overset{\cdot }{\varepsilon }$) had monotonous characters with maxima that are typical enough for the superplastic behavior of the fine-grained alloys (see [24,25,26,27,28,29,47,48,49]). At the strain rate $\overset{\cdot }{\varepsilon }$ = 10^−2^ s^−1^, the maximum values of *δ_max_* for the majority of UFG Al-0.5%Mg-Sc alloys were achieved at 450 °C.

As one can see from Table 2, the preliminary annealing resulted in a decrease of the yield stress of the UFG alloys regardless of the Sc content as well as to the test temperature and strain rate. The largest decrease of the yield stress was observed in the case of strain at 300 and 350 °C. The strain in the temperature range 450–500 °C, the differences between the values in *σ_b_* between the non-annealed and annealed specimens did not exceed 2–3 MPa.

The effect of preliminary annealing on the plasticity of the UFG alloys had a more complex character. As one can see from Table 2, the preliminary annealing did not affect the elongation to failure considerably when testing at 300–350 °C. However, it resulted in some decreasing of plasticity of the UFG Al-0.5%Mg-Sc alloys at elevated test temperatures (450 and 500 °C) and at increased strain rates (from 10^−1^ s^−1^ and higher).

**Figure 13:**
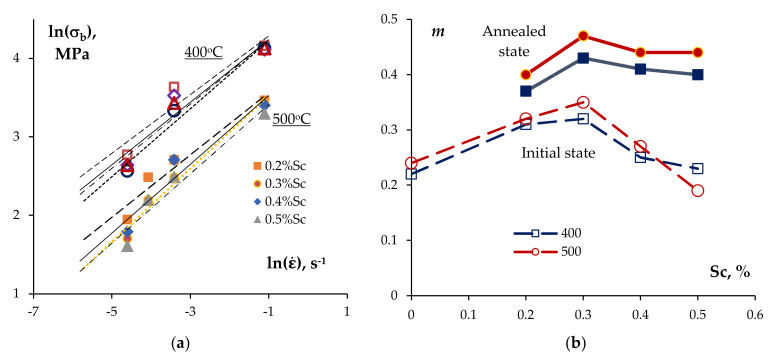
Analysis of the superplasticity test results for the UFG Al-0.5%Mg-Sc alloy specimens: (**a**) dependencies of the yield stress on the strain rate in the logarithmic axes ln($\sigma_{b}$) − ln$\left(\overset{\cdot }{\varepsilon }\right)$; (**b**) dependencies of the strain rate sensitivity coefficient $m=\ln\left(\sigma_{b}\right)/\ln\left(\overset{\cdot }{\varepsilon }\right)$ on the Sc concentration: test temperature—400 °C (squares) and 500 °C (circles); empty symbols–initial state [30]; full symbols–annealed state.

The values of the strain rate sensitivity coefficient *m* were calculated from the slopes of the dependencies $\sigma_{b}\left(\overset{\cdot }{\varepsilon }\right)$, which can be interpolated by a straight line in the logarithmic axes ln($\sigma_{b}$) − ln$\left(\overset{\cdot }{\varepsilon }\right)$ with good accuracy (Figure 13a). Figure 13b presents the dependencies of the strain rate sensitivity coefficient $m=\ln\left(\sigma_{b}\right)/\ln\left(\overset{\cdot }{\varepsilon }\right)$ on the Sc concentration. The analysis of the results obtained has shown the magnitude of the coefficient *m* in the annealed alloys at the test temperatures 400 °C and 450 °C to be 0.37–0.43 and 0.40–0.47, respectively. The values of coefficient *m* in the annealed alloys were slightly higher than the ones in the non-annealed alloys. Note also that no essential decreasing of *m* was observed at high Sc concentrations (0.4–0.5%) (Figure 13b).

**Figure 14:**
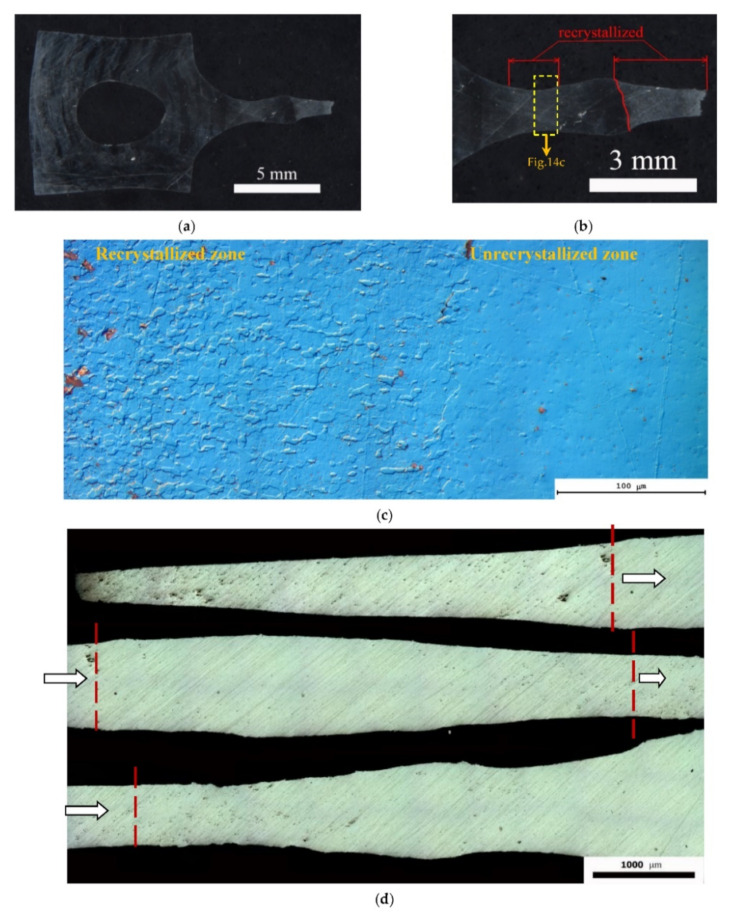
General view of the deformed parts of the UFG alloy specimens after the tension tests (500 °C, 10^−2^ s^−1^). The area of the plastic deformation localization: (**a**–**c**) Al-0.5%Mg-0.2%Sc; (**d**) Al-0.5%Mg-0.3%Sc.

The metallographic investigations of the destroyed specimen surfaces evidenced an intensive pore formation during the superplasticity testing of the UFG alloys (Figure 14d). The largest pores are formed in the destruction region as well as within the localized deformation areas. Note also that the mean pore sizes in the deformed parts of the UFG alloy specimens were smaller than the ones in the destroyed parts of the cast alloy specimens. In the metallographic studies, in the case of the use of the same magnifications, it was manifested visually in several specimens as a decreasing of the volume fraction of pores.

The results of fractographic analysis have shown the fractures of all UFG alloy specimens after the superplasticity testing to have a viscous character. These can be described as a set of pits of various sizes (Figure 11). Variations of test temperature and strain rate did not affect the fracture character considerably.

### 3.3. Dynamic Grain Growth

The metallographic and electron microscopy investigations conducted have shown no essential changes in the microstructure of the cast Al-0.5%Mg-Sc alloys during the superplastic deformation. The dependence of the microhardness on the heating temperature had a two-stage character with a maximum similar qualitatively to the one of the dependence of the microhardness on the 30-min annealing temperature (see [30,38]). It allows suggesting the character of the microhardness changes in the cast alloys with increasing test temperature to be determined by nucleation and growth of the Al_3_Sc particles. The microhardness values for the deformed areas and for the non-deformed ones differ no more than in 50–60 MPa. The maximum values of the microhardness in the alloy with the maximum Sc content (0.5%) were 600–610 MPa that is ~1.5 times higher than the ones of the cast Al-0.5%Mg-0.5%Sc alloy in the initial state (400 MPa, see [38]). However, these values appeared to be lower than the maximum microhardness values (~900 MPa, see [38]) in the cast Al-0.5%Mg-0.5%Sc alloy after annealing at 350–375 °C for 30 min. Lower values of microhardness of the cast specimens after the tensile testing, in our opinion, are caused by differences in the heating times. In the case of the tensile testing at 400 °C with the rate of 10^−2^ s^−1^, it was <15 min (taking into account the 10-min holding in the furnace prior to the start of testing).

Prior to describing the results of investigations of the dynamic grain growth in the UFG alloys, it is worth noting that in the specimens deformed in the conditions close to the optimal ones for the superplasticity, clearly expressed plastic deformation localization areas (so called “neckings”) were observed. A typical view of a UFG alloy specimen with the deformation localization areas is presented in Figure 14. Electron microscopy and metallographic investigations have shown the recrystallization processes inside the areas of plastic deformation localization and outside the ones to be different. The volume fraction of the recrystallized structure (*f_R_*) inside the areas of plastic deformation localization was very high whereas outside the ones f_R_ did not exceed 10–15%. Additionally, it is interesting to note that an increased porosity was observed in the areas of plastic deformation localization in some specimens despite the quite large distance from the destruction areas (Figure 14c). It should be stressed that the effect of plastic deformation localization was observed also in the non-annealed specimens of the UFG Al-0.5%Mg-Sc alloys (see Figure 7a in [30]). However, the degree of deformation localization (the magnitude of specimen thinning) was lower considerably.

Next, we studied the microstructure parameters (the volume fraction of the recrystallized structure *f_R_* and the mean sizes of the recrystallized grains *d*) and the microhardness in the regions, the size of which did not exceed 0.5–1 mm from the destruction points.

It should be stressed here that the preliminary annealing at 300 °C resulted in the stabilization of the microstructure of the UFG Al-0.5%Mg-Sc alloys. The volume fraction of the recrystallized structure in the non-annealed specimens exceeded 80% after the superplasticity testing at 300 and 350 °C. At elevated test temperatures (450, 500 °C), the whole deformed parts of specimens were recrystallized almost completely. As one can see from Table 3 after the superplasticity testing, considerably smaller volume fractions of the recrystallized structure were observed in the specimens annealed in advance as compared to the non-annealed specimens. In the non-deformed areas of the UFG Al-0.5%Mg-0.2%Sc alloy specimens, the volume fraction of the recrystallized structure did not exceed 10% even after testing at 500 °C. The maximum volume fraction of the recrystallized microstructure (*f_R_*~80%) in the deformed part of the UFG alloy Al-0.5%Mg-0.2%Sc specimen was observed after testing at 500 °C with the strain rate 10^−2^ s^−1^ (Table 3).

The increase of the strain rate resulted in a decrease in the volume fraction of the recrystallized structure and of the mean grain sizes in the deformed region d_2_ (Table 3). The dependence of the volume fraction of the recrystallized structure (*f_R_*) on the heating time can be described using the Avrami equation: *f_R_* = 1− exp(−*t*/*τ*)*^n^* where *n* is a numerical coefficient and *τ* is the characteristic time of the diffusion-controlled process, which, in the first approximation, can be described by the equation: *τ* = *τ*_0_⋅exp(*Q*/*kT*). A similar equation is used often to describe the dependence of *f_R_* on the strain degree (*ε*): *f_R_* = 1 − exp(−B·*ε*)*^m^* where *m* and *B* are some numerical parameters. In most cases, the increase of the strain rate would lead to the decreasing of the tensile time (*t*) and to the decreasing of the degree of elongation to failure (*δ*) (see Table 2). In this connection, one can consider the dependence of the volume fraction of the recrystallized structure on the strain rate *f_R_*($\overset{\cdot }{\varepsilon }$) to be described well (in the first approximation) by the Avrami equation.

**Figure 15:**
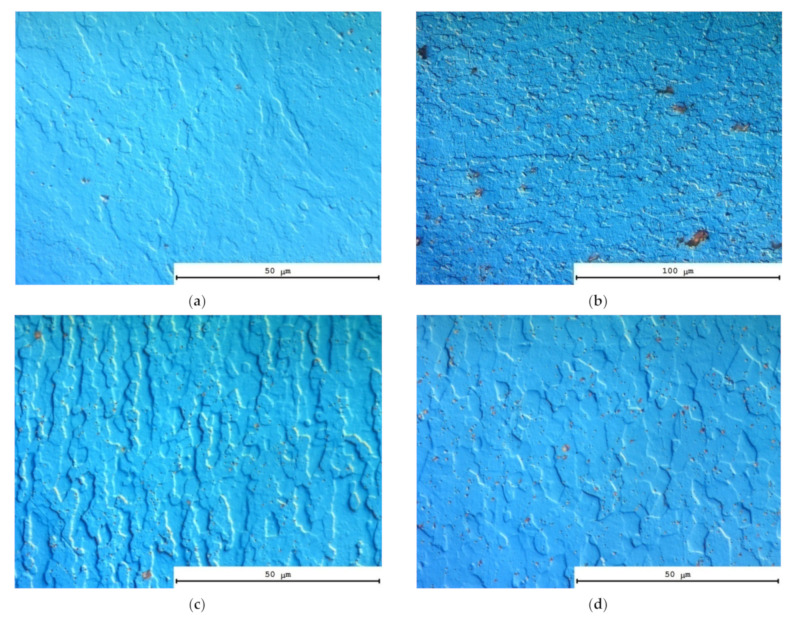

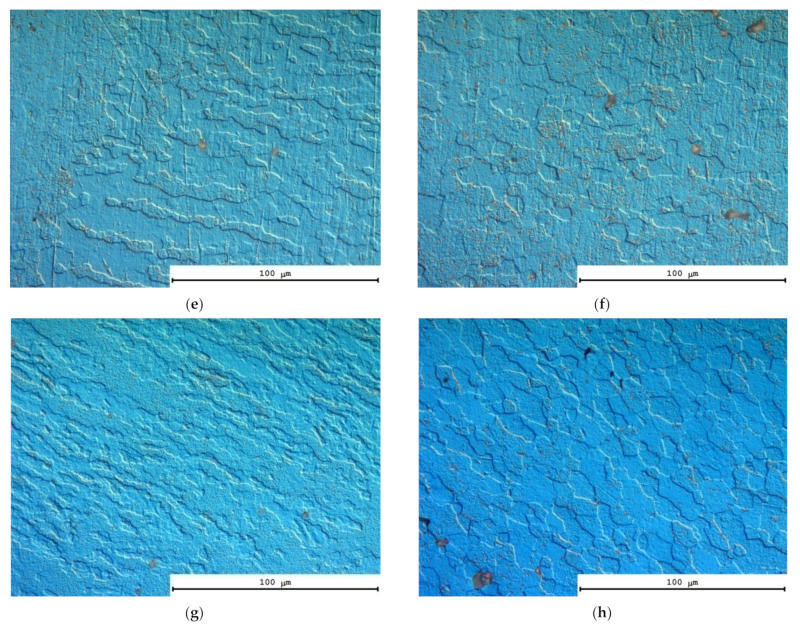
Microstructure of the non-deformed (**a**,**c**,**e**) and deformed (**b**,**d**,**f**) parts of the UFG alloy specimens Al-0.5%Mg-0.2%Sc (**a**–**f**) and Al-0.5%Mg-0.2%Sc (**g**,**h**) after the tension tests. Strain rate 10^−2^ s^−1^. Test temperature 350 °C (**a**,**b**); 400 °C (**c**,**d**), 500 °C (**e**–**h**).

**Figure 16:**
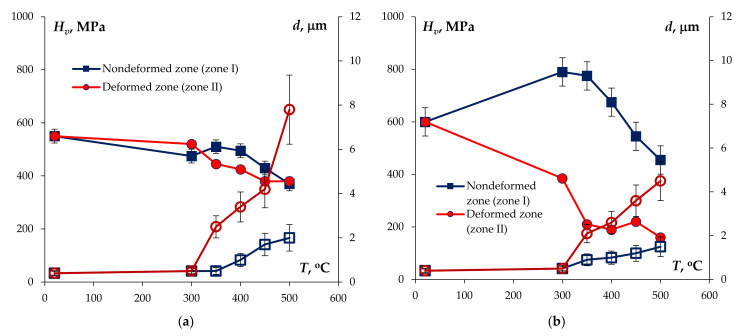
Dependencies of the mean grain sizes (empty symbols) and of the microhardness (full symbols) in the non-deformed (1) and deformed (2) parts on the test temperature and strain rate: UFG alloys with 0.2%Sc (**a**) and 0.3%Sc (**b**).

Figure 15 presents the images of the recrystallized grain microstructure of the UFG Al-0.5%Mg-Sc alloys in the deformed regions and in the non-deformed ones after tension testing. As one can see in Figure 15, intensive dynamic grain growth takes place during superplastic deformation. The mean grain sizes in the deformed regions (*d*_2_) exceeded the ones in the non-deformed regions (*d*_1_). The values of d_1_ and d_2_ are presented in Table 3. In Figure 16, the dependencies of *d*_1_ and *d*_2_ on the test temperature are presented. As one can see from Table 3 and Figure 16, an increase in the mean grain size and of the volume fraction of the recrystallized microstructure with increasing test temperature was observed.

The increasing of the Sc content resulted in a decreasing of the volume fraction of the recrystallized structure and of the mean grain sizes in both deformed and non-deformed parts of the specimens. As one can see from Table 3, in the UFG alloys with 0.3–0.5%Sc, the volume fraction of the recrystallized microstructure of the non-deformed parts of the specimens was 1% or less. The mean grain sizes were close to the initial ones.

The analysis of the dependencies of the microhardness on the test temperature presented in Figure 16 shows the microhardness in the deformed parts of the specimens to be considerably lower than the one in the non-deformed parts. In our opinion, it is related to the intensive dynamic grain growth in the deformed parts of the UFG alloy specimens, which leads to the mean grain sizes *d*_2_ to be considerably higher than the ones in the non-deformed parts (*d*_1_).

**Figure 17:**
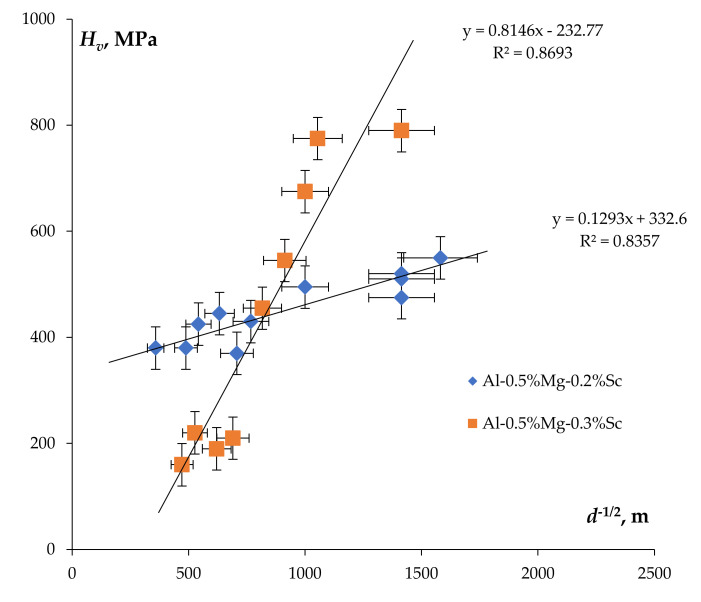
Dependence of the microhardness on the grain size in the *H_v_* − *d*^−1/2^ axes. Analysis of the results is presented in Figure 12.

The dependencies of the microhardness on the mean grain size for the deformed parts of the UFG Al-0.5%Mg-Sc alloy specimens can be described with good accuracy using the Hall–Petch equation: $H_{v}=H_{v0}+K/\sqrt{d}$ where *H_v_*_0_ is the microhardness of the crystal lattice, *K* is the grain boundary hardening coefficient (Hall–Petch coefficient). One can see in Figure 17 that the dependencies *H_v_*(*d*) in the *H_v_* − *d*^−1/2^ axes can be interpolated by straight lines with a satisfactory accuracy (for the majority of alloys, the reliability of the linear approximation R^2^ > 0.8). The magnitude of the coefficient *K* for the annealed alloys increased with increasing Sc concentration in the UFG alloys and was close to the values of parameter *K* in the non-annealed UFG alloys (see [38]).

It is interesting to note that higher values of the coefficient *K* were observed in the alloys with increased Sc content. The formation of the second phase (Al_3_Sc) particles at the grain boundaries would dampen the crossing of the grain boundaries by the dislocation bunches and make the functioning of the Frank–Reed source in the adjacent grains difficult. To explain this effect, the model described in [50] can be used also. According to this model, the dislocation loops may form around the non-coherent second phase particles (see also [51,52,53,54,55]). At the same time, it is worth noting that the negative values of the coefficient *H_v_*_0_ for the UFG alloys with increased Sc content (see Figure 17) were unexpected since the nucleation of the Al_3_Sc particles was expected to result in an increase of *H_v_*_0_ (see [50,55]). The analysis of the nature of this effect in the dynamic grain growth in the superplasticity conditions will be continued in our further studies.

## 4. Discussion

The mechanisms of superplastic deformation of the UFG Al-0.5Mg-Sc alloys were described in [30]. It should be stressed only that the high values of the strain rate sensitivity coefficient (*m* = 0.40–0.47 at the test temperature 450 °C, see Figure 13b) evidence for the grain boundary sliding to be the primary mechanism of the high-temperature deformation of the UFG alloys. The equiaxial shapes of the grains in the destruction zone (Figure 11) are also indirect sign evidence in favor of this suggestion. In the coarse-grained Al alloys, the primary mechanism of the high-temperature plastic deformation is the power-law creep [47,48], the strain rate of which is much lower than the one of the grain boundary sliding in the UFG alloys. The difference in the deformation mechanisms in the cast and UFG alloys resulted in the differences in the values of the relative elongation to failure for the cast and UFG alloys (see Table 1 and Table 2). Different characters of the effect of the test temperature on the elongation for the cast and UFG Al-0.5%Mg-Sc alloys are evidence in favor of this suggestion indirectly.

Let us analyze the effect of preliminary annealing on the ultimate characteristics of the superplastic deformation and on the kinetics of the dynamic grain growth in the UFG Al-0.5%Mg-Sc alloys.

Note that the goal of the preliminary annealing at 300 °C was the nucleation of the Al_3_Sc particles providing the stabilization of the nonequilibrium microstructure in the UFG Al-0.5%Mg-Sc alloys. Therefore, preliminary annealing was expected to allow forming smaller grains in the UFG alloys, decreasing the intensity of the dynamic grain growth, and, as a consequence, improving the plasticity of the alloys at elevated test temperatures. As one can see from Table 2, this effect was not achieved–the elongation to failure of the annealed UFG alloys differed from the magnitudes of *δ* for the non-annealed alloys insufficiently.

In our opinion, there are at least two reasons why the increased plasticity of the UFG Al-0.5%Mg-Sc alloys was not achieved.

The main reason is that the ultimate elongation to failure in the UFG Al-0.5%Mg-Sc alloys is controlled likely by the pore formation at the large Al_3_Sc particles (see [30] as well as Introduction). The preliminary annealing leads to the nucleation of the Al_3_Sc particles, in particular, to the formation of large, elongated particles near the grain boundaries via the discontinuous precipitation mechanism (Figure 6). These large particles are the points of formation and growth of the pores and, as a consequence, promote the cavitation destruction of the UFG Al-0.5%Mg-Sc alloy specimens. According to the model [41,42], for the initiation of a micropore, the power of the disclination loop forming at Al_3_Sc particle during the superplastic deformation should reach its critical value ω*. The magnitude of the critical power of a disclination loop can be calculated easily from the equality of the energy of disclination and the one of the free surface of a pore or a crack of a given size. Accordingly, one can expect the disclination loops forming at the large Al_3_Sc particles to reach the critical value ω* faster. So far, the nucleation of the large Al_3_Sc particles in the course of preliminary annealing provides the conditions limiting the maximum plasticity of the UFG alloys. To ensure higher ultimate characteristics of superplasticity, one should minimize the volume fraction of the Al_3_Sc particles forming via the discontinuous precipitation mechanism.

The second factor, which does not allow providing the improved superplastic characteristics of the UFG alloys is the specifics of the dynamic grain growth in the annealed UFG Al-0.5%Mg-Sc alloys. As it has been shown above, the annealed UFG alloys are featured by an increased tendency to the plastic deformation localization at the microscopic level (Figure 1 and Figure 14).

The origin (or the origins) of the effect of the preliminary annealing on the character of the plastic deformation localization in the UFG Al-0.5%Mg-Sc alloys are not clear at the moment and additional investigations are necessary. Can be one of the possible origins of this the presence of the residual dendrite macrostructure, which was manifested in the metallographic investigations of the non-deformed parts of the specimens (Figure 1). The nucleation of the Al_3_Sc particles at the dendrite boundaries damping the motion of dislocations, in our opinion, may promote the enhanced tendency of the Al-0.5%Mg-Sc alloys to the plastic deformation localization. We suppose that the disappearance of the uniform plastic flow stage in the annealed cast Al-0.5%Mg-Sc alloy specimens is also evidence in favor of this assumption indirectly. Note also that the characteristic distance between the macro-neckings of the plastic deformation localization (Figure 14) was close to the one between the dendrite boundaries, which was 0.3–1 mm (Figure 1). In our opinion, it also evidences an important role of the dendrite macrostructure in the manifestation of the plastic deformation localization in the UFG Al-0.5%Mg-Sc alloys. In order to increase the uniformity of the plastic flow of the UFG Al-0.5%Mg-Sc alloys at the macroscopic level, it is necessary to apply the technologies of preliminary hot deformation processing allowing removing the dendrite nonuniformity macrostructures completely.

**Figure 18:**
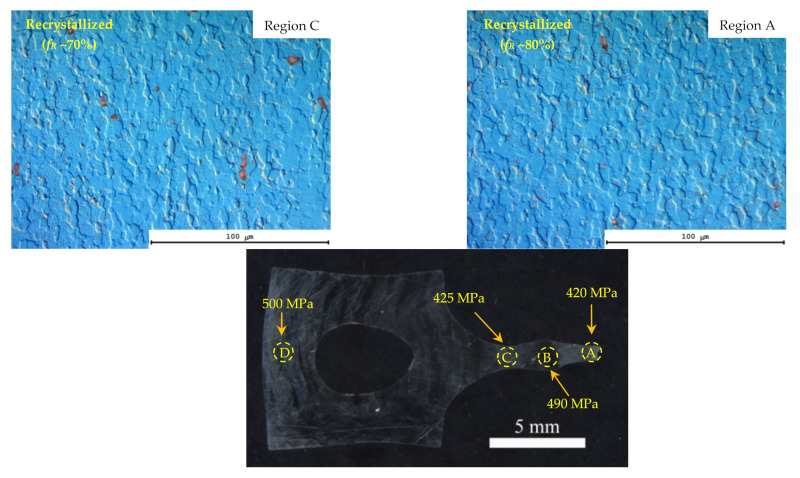

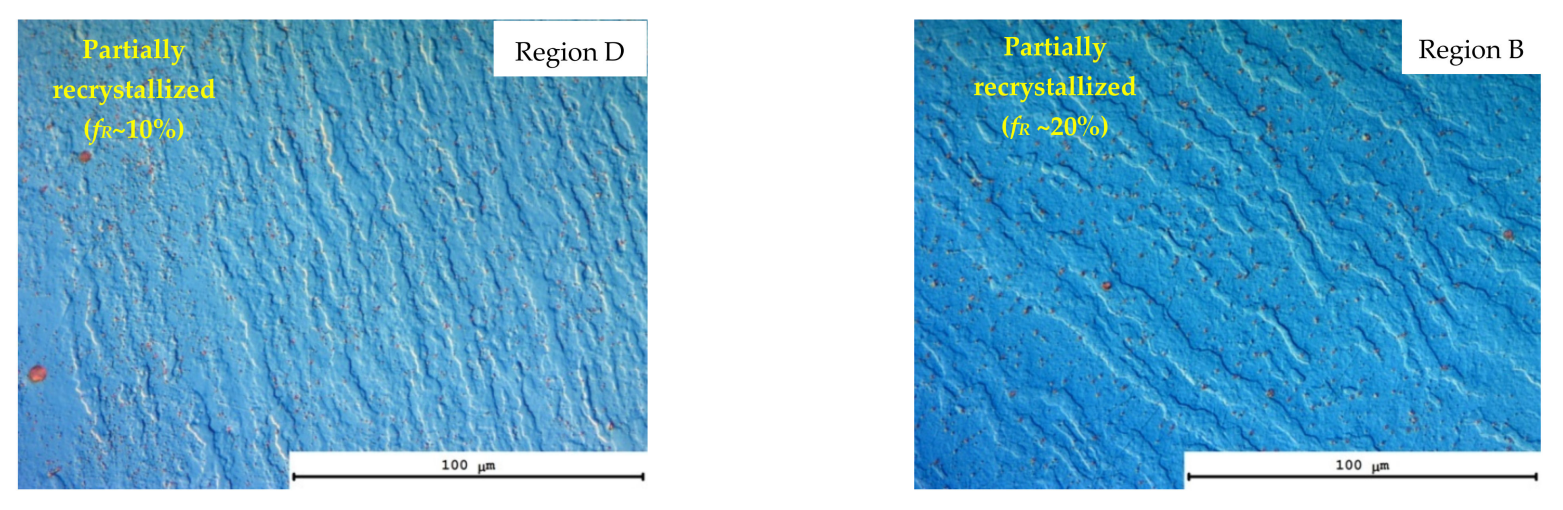
Results of the microhardness and microstructure investigations in different areas of the deformed part of the UFG Al-0.5%Mg-0.2%Sc alloy specimen after the tension tests at the temperature 450 °C and strain rate 10^−2^ s^−1^.

As it has been shown above, the macrolocalization of the plastic deformation leads to a nonuniformity of recrystallization inside the specimens. The presence of the regions with different grain sizes in the structure of the material and, as a consequence, with different values of hardness (Figure 18) suppresses the possibility of uniform plastic flow of the material. It is a negative factor, which should be taken into account when selecting the optimal regimes of fabricating the small-sized wires using the hot deformation method (drawing, rolling, extraction, etc.).

Let us analyze the kinetics of the dynamic grain growth during the superplastic deformation of the UFG Al-0.5%Mg-Sc alloys. To describe the dynamic grain growth in the UFG Al-0.5%Mg-Sc alloys, we will use the approach developed earlier within the framework of the theory of structural superplasticity [56,57] and of the theory of nonequilibrium grain boundaries in fine-grained metals [58]. Within the framework of this approach, the grain growth rate in the UFG materials is governed by the defects at the grain boundaries, which generate the long-range internal stress fields *σ_i_*. According to [56,58], the interactions of the defects distributed inside the grain boundary with the fields of the external stress (*σ*) and of the internal stress (*σ_i_*) result in the arising of additional driving forces for the grain boundary migration:

(1) $$P=\left(\sigma +\sigma_{i}\right)\left(\rho_{b}\Delta b+\omega \right)=\bar{\sigma }\left(\rho_{b}\Delta b+\omega \right)=P_{\rho }+P_{\omega }$$

where *σ_i_* is the internal stress field generated by the defects distributed inside the grain boundaries and in the triple joints of the ones:

(2) $$\sigma_{i}=\alpha_{1}G\rho_{b}^{st}\Delta b+\alpha_{2}G\omega$$

Here *G* is the shear modulus, $\rho_{b}^{st}$ is the stationary density of the orientation mismatch dislocations (OMDs) in the non-equilibrium grain boundaries in the UFG metal, Δ*b* is the Burgers vector of OMD, *α_1_* and *α_2_* are the numerical coefficients.

Besides, the defects affect the diffusion mobility of the grain boundaries *M*. At high power of the disclination dipoles, these ones can limit the mobility [58]:

(3) $$M^{-1}=M_{b}^{-1}+M_{\rho }^{-1}+M_{\omega }^{-1}$$

where *M_b_* is the mobility coefficient of a defectless grain boundary, *M_ρ_* is the mobility coefficient of the OMDs distributed inside grain boundary, and *M_ω_* is the mobility coefficient of the joint disclinations [58]. The values of contributions *M_b_*, *M_ρ_*, and *M_ω_* can be calculated using the following formulas [58]:

(4) $$M_{\rho }=A_{\rho }C_{b}\left(b/d\right)\left(b/G\right)\frac{1}{\rho_{b}^{st}\Delta b},\;M_{\omega }=A_{\omega }C_{b}{\left(b/d\omega \right)}^{2}\left(b/G\right),\;M_{b}=A_{b}C_{b}{\left(b/d\right)}^{2}\left(b/G\right),$$

where $C_{b}=G\delta D_{b}/kT$, $A_{\rho }=2\pi /\ln\left(d/b\right)$, $A_{\omega }\sim 1$, $A_{b}=\left(5V_{m}/\Delta b\right){\left(d_{0}/b\right)}^{4}\left(Gb/\gamma_{b}\right)$, *V_m_* is the grain growth rate during annealing measured experimentally, *δ* = 2*b* is the grain boundary width, *b* is the Burgers vector, *D_b_* is the grain boundary diffusion coefficient, *k* is the Boltzmann constant, and *γ_b_* is the grain boundary energy.

The grain growth rate *V_m_* can be related to the effective migration mobility *M* and to the driving force *P* by usual relation [58]:

(5) $$V_{g}=MP$$

At low power of the joint disclinations, the mobility of the grain boundaries is determined by the mobility of the OMDs, and the driving force is related to the interaction of these ones with the external stress field [56,58]:

(6) $$V_{g}=\overset{\cdot }{d}=A_{\rho }C_{b}\left(\sigma /G\right)\left(b/d\right)b$$

In the case of high *ω*, the mobility of the grain boundaries in the superplasticity regime is governed by the mobility of the disclination dipoles *M_ω_*, ant the driving force is related to the interaction of the disclination dipoles with each other [56,57]:

(7) $$V_{g}=\overset{\cdot }{d}=A_{\omega }C_{b}{\left(b/d\right)}^{2}b$$

In the intermediate case, the dynamic grain growth rate can be written in more general form:

(8) $$\overset{\cdot }{d}=A{\left(d/b\right)}^{-x}$$

where $A≅C_{b}b{\left(\sigma /G\right)}^{2-x}$ and the magnitude of the exponent *x* takes the values from 1 to 2 subject to the joint disclination power *ω*. At low *ω*, *x* = 1, and $A=A_{\rho }C_{b}b\left(\sigma /G\right)$. At high *ω*, *x* = 2, and $A=A_{\omega }C_{b}$.

The efficiency of application of the theoretical models described in [56,57,58] has been demonstrated earlier when describing the dynamic grain growth in the non-annealed UFG Al-0.5%Mg-Sc alloys (see [30]).

**Figure 19:**
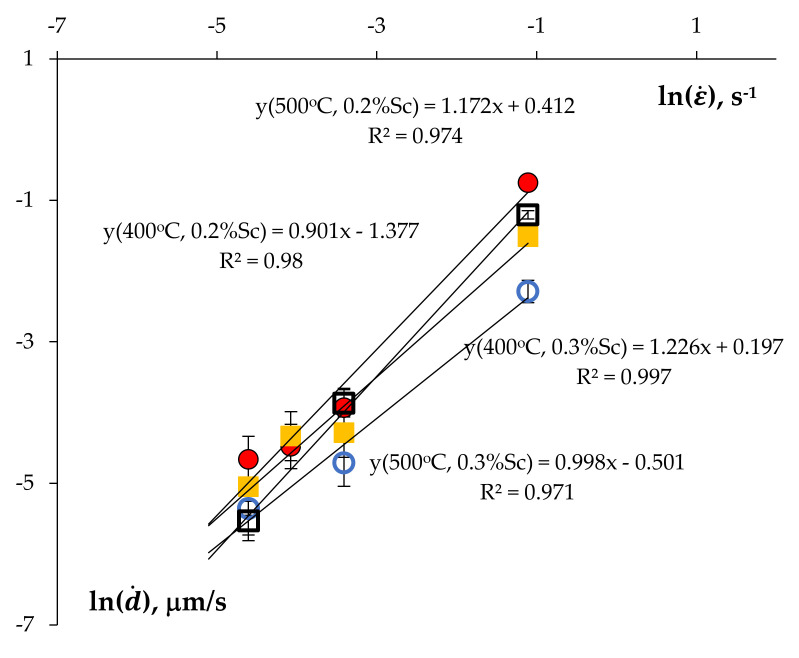
Dependence of the dynamic grain growth rate on the strain rate in the logarithmic axes. UFG alloys with 0.2%Sc (circles) and 0.3%Sc (squares). Test temperature 400 °C (empty markers) and 500 °C (full markers).

According to [47,56,57], usually, the dependence of the grain growth rate on the strain rate measured experimentally is expressed in the form $\overset{\cdot }{d}\sim {\overset{\cdot }{\varepsilon }}^{k}$ where the parameter *k* depends on the strain rate $\overset{\cdot }{\varepsilon }$ can be determined from the slope of the curve lg($\overset{\cdot }{d}$) − lg($\overset{\cdot }{\varepsilon }$) at fixed values of the strain degree. As one can see in Figure 19, the measured magnitude of the coefficient *k*_exp_ for the UFG Al-0.5%Mg-Sc alloys at 400 °C and 500 °C varied from ~0.9 to ~1.2. The calculated values of *k*_exp_ agree well with the values for the non-annealed UFG Al-0.5%Mg-Sc alloys (see Figure 16 in [30]). Comparing the values of *k*_exp_ with the theoretical ones *k_th_* presented in [30] shows the kinetics of the dynamic grain growth in the annealed UFG alloys to be governed by the mobility of the OMDs.

## 5. Conclusions

1. The superplasticity of the cast and ultrafine-grained (UFG) Al-0.5%Mg-Sc alloys with the Sc contents from 0.2 to 0.5 wt.% has been studied. The cast structure in the alloys was formed by induction casting without application of subsequent homogenization. The UFG structure was formed by ECAP. The stabilization of the nonequilibrium UFG structure was provided by preliminary annealing at 300 °C that did not exceed the recrystallization temperature in the investigated alloys. In the course of preliminary annealing, the Al_3_Sc particles of two types, nucleated–coherent Al_3_Sc nanoparticles inside the grains and relatively large (50–200 nm) elongated fan-shaped Al_3_Sc particles formed via the discontinuous decay mechanism.
2. The UFG alloys have good superplastic characteristics–in the annealed UFG Al-0.5%Mg-0.5%Sc alloy, the relative elongation to failure reached 900% (test temperature 500 °C, strain rate 3.3 × 10^−2^ s^−1^). The magnitude of strain rate sensitivity coefficient *m* was 0.4–0.47. At reduced test temperatures (300–350 °C) not exceeding the recrystallization temperature, the elongation to failure in the annealed UFG alloys varied from 170% to 320%.
3. The values of the elongation to failure for the annealed UFG Al-0.5%Mg-Sc alloys are comparable to the ones for the non-annealed alloys tested in the same temperature and rate strain conditions. The close values of elongation in the UFG alloys with different grain sizes are caused likely by the following factors:
  (a) The formation of pores at large Al_3_Sc particles forming via the discontinuous decay mechanism during preliminary low-temperature annealing. The generation and growth of the pores at the large Al_3_Sc particles leads to accelerated cavitation destruction of the UFG Al-0.5%Mg-Sc alloys.
  (b) Nonuniformity of the plastic deformation at the macroscopic level and the formation of the macro-neckings of the localized plastic deformation. The low-temperature annealing leads to the increase of the macro-localization scale during the superplastic deformation of the UFG Al-0.5%Mg-Sc alloys.
  (c) Accelerated dynamic grain growth, the kinetics of which is determined by the mobility of the orientation mismatch dislocations in the non-equilibrium grain boundaries in the UFG alloys.

## Figures and Tables

**Table 1:** Results of the superplasticity tests of the coarse-grained cast alloys.

*T*, °C	$\overset{\cdot }{\varepsilon }$, s^−1^	Sc Concentration in the Alloy, wt.%											
		0.3%						0.5%					
		Initial State		Annealing 300 °C, 10 h		Annealing 300 °C, 300 h		Initial State		Annealing 300 °C, 10 h		Annealing 300 °C, 300 h	
		*σ_b_*, MPa	*δ*, %	*σ_b_*, MPa	*δ*, %	*σ_b_*, MPa	*δ*, %	*σ_b_*, MPa	*δ*, %	*σ_b_*, MPa	*δ*, %	*σ_b_*, MPa	*δ*, %
300	10^−2^	43	53	55	56	55	90	58	66	70	85	64	90
350	10^−2^	32	51	38	38	37	46	50	68	52	75	50	68
400	10^−2^	21	51	26	52	31	46	36	66	38	80	45	42
	3.3 × 10^−2^	-	-	40	90	40	52	46	76	52	100	54	87
	3.3 × 10^−1^	43	69	54	90	46	85	63	77	70	115	70	110
450	10^−2^	15	45	21	50	25	30	29	50	31	70	34	30
500	10^−2^	11	24	18	30	17	33	22	43	25	58	29	70
	1.7 × 10^−2^	-	-	-	-	22	60	-	-	-	-	33	90
	3.3 × 10^−2^	18	46	22	50	24	53	26	55	29	55	33	50
	3.3 × 10^−1^	-	-	-	-	28	70	36	80	-	-	40	75

**Table 2:** Results of the superplasticity tests of the UFG alloys.

*T*, °C	$\overset{\cdot }{\varepsilon }$, s^−1^	Sc Concentration in the Alloy, wt.%															
		0.2%				0.3%				0.4%				0.5%			
		Initial State [30]		Annealing 300 °C, 300 h		Initial State [30]		Annealing 300 °C, 1 h		Initial State [30]		Annealing 300 °C, 300 h		Initial State [30]		Annealing 300 °C, 1 h	
		*σ_b_*, MPa	*δ*, %	*σ_b_*, MPa	*δ*, %	*σ_b_*, MPa	*δ*, %	*σ_b_*, MPa	*δ*, %	*σ_b_*, MPa	*δ*, %	*σ_b_*, MPa	*δ*, %	*σ_b_*, MPa	*δ*, %	*σ_b_*, MPa	*δ*, %
300	10^−2^	65	160	44	170	59	205	44	250	56	225	48	200	57	345	42	275
350	10^−2^	33	290	28	235	32	295	20	320	28	280	28	300	33	320	22	250
400	3.3 × 10^−3^	9	275	-	-	7	510	-	-	10	250	-	-	11	290	-	-
	10^−2^	10	560	15	360	12	490	13	480	12	350	14	460	13	260	13	370
	3.3 × 10^−2^	20	350	39	220	18	425	28	300	17	460	35	280	21	480	32	300
	10^−1^	24	290	-	-	20	360	-	-	23	320	-	-	23	680	-	-
	3.3 × 10^−1^	-	-	65	130	-	-	62	220	-	-	60	150	-	-	62	130
450	10^−2^	13	350	10	500	10	490	9	350	10	500	9	440	14	400	8	420
500	3.3 × 10^−3^	4	400	-	-	4	540	-	-	4	330	-	-	4	520	-	
	10^−2^	8	265	7	360	5	820	5.5	700	4	530	6	420	5	670	5	320
	1.7 × 10^−2^	-	-	12	330	-	-	9	440	-	-	9	380	-	-	9	500
	3.3 × 10^−2^	9	350	15	370	6	625	12	720	8	840	15	580	7	750	12	900
	10^−1^	14	220	-	-	13	400	-	-	7	1060	-	-	9	1055	-	-
	3.3 × 10^−1^	-	-	32	175	-	-	31	330	15	500	30	240	-	-	26	410

**Table 3:** Results of investigations of the dynamic grain growth in the specimens of Al-0.5%Mg-Sc alloys after tensile testing ^1,2^.

*T*, °C	$\overset{\cdot }{\varepsilon }$, s^−1^	Sc Concentration in the Alloy, wt.%															
		0.2%				0.3%				0.4%				0.5%			
		Initial State [30]		Annealing 300 °C, 300 h		Initial State [30]		Annealing 300 °C, 1 h		Initial State [30]		Annealing 300 °C, 300 h		Initial State [30]		Annealing 300 °C, 1 h	
		*d*_1_, μm ^1^	*d*_2_, μm ^1^	*d*_1_, μm (*f_R_*,%)	*d*_2_, μm (*f_R_*,%)	*d*_1_, μm	*d*_2_, μm	*d*_1_, μm (*f_R_*,%)	*d*_2_, μm (*f_R_*,%)	*d*_1_, μm	*d*_2_, μm	*d*_1_, μm (*f_R_*,%)	*d*_2_, μm (*f_R_*,%)	*d*_1_, μm	*d*_2_, μm	*d*_1_, μm (*f_R_*,%)	*d*_2_, μm (*f_R_*,%)
20	10^−2^	0.4–0.5		0.5–0.8		0.4–0.5		0.5–0.7		0.4–0.5		0.5–0.7		0.4–0.5		0.5–0.7	
300	10^−2^	0.4–0.5	0.4–0.5	0.5–0.8 (<1)	0.5–0.8 (<1)	0.4–0.5	0.4–0.5	0.5–0.8 (0)	2.3 (<1)	0.4–0.5	1.5	0.5–0.7 (0)	0.5–0.7 (<1)	0.4–0.5	0.4–0.5	0.5–0.7 (0)	0.5–0.7 (0)
350	10^−2^	0.4–0.5	2.5	1.6 (<10)	3.4 (55)	0.4–0.5	2.1	0.5–0.8 (0)	2.5 (35)	0.4–0.5	2.0	0.5–0.7 (0)	1.9 (45)	0.4–0.5	1.9	0.5–0.7 (0)	1–1.5 (<10)
400	3.3 × 10^−3^	1.5	4.8	-	-	1.3	3.3	-	-	1.2	3.1	-	-	1.2	3.1	-	-
	10^−2^	1.3	4.1	-	-	1.2	2.9	-	-	1–1.2	2.9	-	-	1–1.2	2.6	0.9 (<1)	1.7–1.9 (<10)
	3.3 × 10^−2^	0.8–1.2	3.4	1.9 (<10)	3.6 (60)	0.8–1	2.7	0.6–0.8 (0)	2.7 (45)	0.5	2.2	0.5–0.7 (0)	2.4 (20)	0.5	2.2	0.5–0.7 (0)	1.5 (<10)
	10^−1^	0.7	2.8	1.7 (<10)	2.3 (15)	0.5	2.6	0.6–0.8 (0)	2.6 (20)	0.5	2.1	0.5–0.7 (0)	2.2 (20)	0.5	2.1	0.5–0.7 (0)	1–1.5 (<10)
	3.3 × 10^−1^	-	-	-	-	-	-	-	-	-	-	-	-	-	-	-	-
450	10^−2^	-	-	1.5 (<10)	1.9 (<10)	-	-	0.6–0.8 (0)	2.6 (<10)	-	-	-	-	-	-	1.5 (<1)	2.3 (20)
500	3.3 × 10^−3^	1.7	4.2	2.0 (<10)	5.0 (70)	1.2	3.6	0.6–0.9 (<1)	2.9 (50)	1.2	2.8	0.5–0.7 (<1)	2.7 (40)	1–1.2	2.4	0.5–0.7 (<1)	2.5 (20)
	10^−2^	2.5	10.3	-	-	2.1	8.0	-	-	1.5	6.0	-	-	1.2	4.5	0.9–2 (<1)	3.8 (20)
	1.7 × 10^−2^	2.2	8.8	-	-	1.7	5.6	-	-	1.2	5.3	-	-	1.2–1.3	3.6	-	-
	3.3 × 10^−2^	2.0	7.8	2.9 (<10)	6.3 (80)	1.5	4.5	0.7–1 (<1)	5.5 (70)	1.0	4.6	0.8–0.9 (<1)	4.8 (60)	1.0	3.5	0.8–0.9 (<1)	4.6 (55)
	10^−1^	-	-	2.6 (<10)	4.8 (30)	-	-	0.7–0.9 (<1)	4.2 (30)	-	-	-	-	-	-	-	-
	3.3 × 10^−1^	0.5	5.6	2 (<10)	4.2 (25)	0.5	3.3	0.6–0.9 (<1)	3.7 (25)	0.5	3.9	0.5–0.7 (<1)	2.9 (25)	0.5	3.2	0.6–1 (<1)	2.7 (20)
		-	-	<1 (<10)	3.5 (<10)	-	-	0.6–0.9 (<1)	2.8 (<10)	-	-	-	-	-	-	-	-

^1^*d*_1_ and *d*_2_ are the average grain sizes in the non-deformed zone (Zone II) of the specimens and in the deformed zone (Zone I) ones after the tensile testing. ^2^ Mean uncertainty of measuring the volume fraction of recrystallized structure was 5 vol.%. The value of *f_R_* < 1% in Table 3 means that few recrystallized grains were identified by metallographic or SEM methods. Mean sizes of these grains are given in the same column. The value *f_R_* = 0% in Table 3 means that we could not identify any recrystallized grains in the investigated areas of the specimens by metallographic or SEM methods.

## Data Availability

Data is contained within the article.
